# Intraoperative Circulatory Support in Lung Transplantation: Current Trend and Its Evidence

**DOI:** 10.3390/life12071005

**Published:** 2022-07-07

**Authors:** Henning Starke, Vera von Dossow, Jan Karsten

**Affiliations:** 1 Institute of Anaesthesiology, Heart and Diabetes Centre NRW, Bad Oeynhausen, Ruhr University Bochum, 44801 Bochum, Germany; hstarke@hdz-nrw.de; 2 Department of Anaesthesiology and Intensive Care Medicine, Hannover Medical School, 30625 Hannover, Germany; karsten.jan@mh-hannover.de

**Keywords:** lung transplantation, circulatory support, extracorporeal membrane oxygenation, diastolic dysfunction, right ventricular failure, reperfusion injury, risk stratification

## Abstract

Lung transplantation has a high risk of haemodynamic complications in a highly vulnerable patient population. The effects on the cardiovascular system of the various underlying end-stage lung diseases also contribute to this risk. Following a literature review and based on our own experience, this review article summarises the current trends and their evidence for intraoperative circulatory support in lung transplantation. Identifiable and partly modifiable risk factors are mentioned and corresponding strategies for treatment are discussed. The approach of first identifying risk factors and then developing an adjusted strategy is presented as the ERSAS (early risk stratification and strategy) concept. Typical haemodynamic complications discussed here include right ventricular failure, diastolic dysfunction caused by left ventricular deconditioning, and reperfusion injury to the transplanted lung. Pre- and intra-operatively detectable risk factors for the occurrence of haemodynamic complications are rare, and the therapeutic strategies applied differ considerably between centres. However, all the mentioned risk factors and treatment strategies can be integrated into clinical treatment algorithms and can influence patient outcome in terms of both mortality and morbidity.

## 1. Introduction

Since it was first performed in 1963, lung transplantation has remained a multidisciplinary challenge, especially in the intraoperative setting [1]. Nevertheless, it is the only therapy for patients with end-stage lung disease for whom drug therapy fails. Advanced lung diseases are often associated with pulmonary arterial hypertension (PAH), resulting in right and, secondarily, left heart failure [2,3,4]. Thus, during transplantation in this highly vulnerable patient population, circulatory support through optimal anaesthesiologic and surgical management plays a crucial role [5,6]. A major change in intraoperative management was the use of extracorporeal membrane oxygenation (ECMO) as a closed system as compared to the cardiopulmonary bypass (CPB). With different cannulation strategies (veno-arterial and veno-venous (VA ECMO and VV ECMO), as well as central and peripheral cannulation), it became possible to support both oxygenation and, if needed, circulation with minimal anticoagulation and with reduced invasiveness [7]. Regarding the application of this procedure, different strategies exist depending on the respective transplant centre. ECMO is used in 15–100% of transplant cases [8,9,10]. In addition to the application of mechanical extracorporeal circulatory support, non-mechanical circulatory support, such as the rational use of catecholamines and inhaled pulmonary vasodilators and the management of right ventricular strain on one lung ventilation (OLV) and diastolic left ventricular dysfunction, are crucial to the success of lung transplantation. The administration of intravenous fluids for circulatory support also has an impact on the management of lung transplantation, including the development of reperfusion injury (RI) [11].

**Figure 1:**
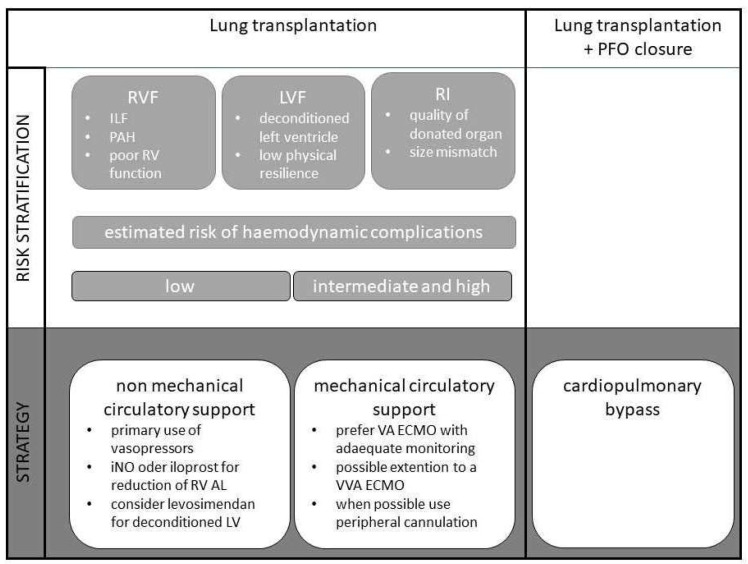
Algorithm for the application of an ERSAS concept for intraoperative circulatory support in lung transplantation.

Irrespective of the clearly differing approaches to circulatory support between individual lung transplant centres, it can make sense to develop strategies for the management of lung transplantation on a patient-specific basis, depending on the risk for certain pathologies, and to define them at an early stage of treatment. Such an approach, for example within the framework of so-called ERSAS concepts (early risk stratification and strategy), aims at identifying possible risk factors for the occurrence of complications and the corresponding strategies and therapies to avoid these complications (Figure 1). This is often and especially used in the perioperative setting.

This review article therefore aims to summarise the current trends of non-mechanical and mechanical circulatory support (MCS) during lung transplantation in an accentuated manner and to reflect the current status of the respective evidence. Focusing on the exemplary but also frequent complications of right ventricular failure (RVF), impairment of left ventricular function (left ventricular failure (LVF)) and RI, the pathophysiology, frequency of occurrence, and known risk factors are first discussed [12,13]. This and possible therapeutic strategies will be discussed on the basis of data from the current literature and especially against the background of the international consensus recommendations for anaesthesiologic and intensive care management of lung transplantation published in 2021 [12,14].

## 2. Risk Stratification

In the context of lung transplantation, the occurrence of various haemodynamic complications is possible. These include RVF, LVF, or left ventricular diastolic dysfunction and RI associated with reperfusion oedema, increased pulmonary vascular resistance, and sterile inflammation [6,12,15].

For none of these entities is there currently a validated scoring system for risk stratification, as is established, for example, in the preoperative risk assessments for the occurrence of cardiac complications in perioperative medicine [16,17]. This is due to the diverse causes for a necessary lung transplantation. In clinical routine, large differences in the operative course can be observed in transplantations due to idiopathic fibrosis with pronounced right heart strain or end-stage chronic obstructive pulmonary disease (COPD) [5]. The analysis of existing databases is also quite difficult, as many centres follow completely different therapeutic strategies during surgery [18]. Hinske et al. developed a score that showed very good predictive power for the need for unplanned mechanical circulatory support. This was achieved in a single-centre study, taking into account the preoperative pulmonary arterial pressure (PAP) and lung allocation scores [19]. However, this risk stratification cannot be used in hospitals that primarily promote the use of ECMO in all transplantations.

The following sections therefore focus on the risk factors that are associated with the occurrence of the respective complications, regardless of chosen therapy strategies (see Table 1).

### 2.1. Right Ventricular Failure

In general, RVF is associated with decreased cardiac output and increased right ventricular filling pressures due to systolic or diastolic dysfunction. In the setting of lung transplantation, pulmonary hypertension is the most important cause of RVF. Other causes, such as pulmonary embolism, myocardial ischaemia, or secondary RVF in left heart failure, are more secondary [20,21].

In patients scheduled for lung transplantation, the incidence of PAH is up to 40–50% [10]. In about 20–30% of transplant candidates, PAH is even the indication for transplantation [9]. It is not uncommon for systemic to suprasystemic blood pressure values to be measured in the pulmonary circulation [9,10].

Pathophysiologically, right heart failure is characterised by dilatation and remodelling of the right ventricle (RV), triggered by an increased right ventricular afterload. Changes in ventricular geometry towards a spherical shape, with displacement of the interventricular septum towards the left ventricle, increased wall stress, and reduced myocardial contractility, are also consequences of the increase in afterload and contribute to the reduction in cardiac output (CO). The decrease in CO is exacerbated by tricuspid regurgitation with corresponding regurgitation volume. Due to reduced left ventricular (LV) filling, ventricular dyssynchrony, and septal kinetic disturbances, the LV is also affected. In advanced stages, end-organ damage such as renal failure, hepatic failure, and intestinal motility disorders can be observed [20,21]. The stress on the right ventricle is further exacerbated by single-lung ventilation. During thoracic surgery, a decrease of RV function up to 25% due to the initiation of OLV and anatomical resection was shown [22]. In principle, the changes in the RV after lung transplantation are reversible and therefore do not represent an absolute contraindication to transplantation even in the case of demonstrable end-organ damage [23].

Predicting the occurrence of acute or acute-on-chronic right heart failure in the setting of lung transplantation is challenging. To our knowledge, no prediction scores exist. Based purely on the intraoperative course of lung transplantation, exacerbation or recurrence of right heart failure is to be expected, primarily before reperfusion of the transplanted lung. Especially in patients with intermediate or severe PAH, immediate positive haemodynamic effects are seen after transplantation. Systolic PAP, mean PAP, and right ventricular end-diastolic volume rapidly decrease. Patients who do not experience these effects are at increased risk for primary graft dysfunction and worse outcomes [24]. Given the link between impaired RV function and outcome, one study investigated the prediction of mortality by echocardiographic parameters in lung transplantation. No pre-transplantation RV parameter predicted all-cause mortality. However, post-transplantation echocardiographic RV parameters (RV strain and systolic PAP) predicted the outcome after lung transplantation [25]. Predicting the occurrence of RVF during lung transplantation also appears difficult because many centres practice liberal use of VA ECMO, which prevents such RVF [10]. Even in centres that favour the selective use of VA ECMO, one of the most important indications for the use of VA ECMO is the existence of severe or intermediate pulmonary hypertension [5,8]. Thus, it is precisely in this risk group that the occurrence of RVF is prevented. Per se, the presence of pulmonary hypertension, idiopathic pulmonary fibrosis, and dilated right ventricle is associated with poor outcomes, even in multivariate analyses [9].

In summary, no clear specific parameters exist to predict RVF during lung transplantation. Patients at particular risk appear to be those who already have severe or intermediate pulmonary hypertension, have pulmonary fibrosis, or do not show recovery of right ventricular function after transplantation.

In 2021, the international consensus recommendations for anaesthesiologic and intensive care management in lung transplantation were published [14]. It was again emphasised that patients with relevant pulmonary hypertension are one of the most challenging patient populations for lung transplantation. The ever-present risk of RVF was also pointed out. However, with weak evidence, no recommendation for a specific risk stratification could be provided here either. It is recommended that a preoperative right heart catheterisation should be performed to assess pulmonary hypertension and right ventricular function. A pragmatic approach is to clinically assess haemodynamic changes and response to inotropics during transplantation after clamping of the pulmonary artery and to derive from this an appropriate risk stratification regarding the occurrence of RVF, and later also to develop an appropriate strategy.

### 2.2. Left Ventricular Failure/Diastolic Dysfunction

The previously described frequent right ventricular dysfunction in patients with chronic lung disease who are scheduled for transplantation results in a decreased left ventricular preload. If prolonged, this results in atrophy of left ventricular cardiomyocytes, which may be associated with both diastolic dysfunction and a reduction in myocardial contractility [26]. This also results in a reduced left ventricular cardiac output and increased filling pressures. Especially in the context of transplantation, the occurrence and extent of pulmonary oedema in the sense of left ventricular congestion can be relevantly increased. Diastolic dysfunction as a so-called deconditioning of the left ventricle is common in patients with PAH. In the total collective of lung transplant candidates, an occurrence of around 30% has been described, depending on the centre, and almost all patients with PAH are affected [9,26]. This also applies to the postoperative course. Therefore, monitoring of LV function is crucial [27].

Porteous et al. investigated the relationship between diastolic left ventricular dysfunction and primary graft dysfunction (PGD) [28]. For the first time, a clear correlation between impaired left ventricular filling and PGD was shown. Correspondingly, evidence between poor outcome and diastolic dysfunction as measured by the ratio between early transmitral flow (E) and mitral annular velocity (e′), which is independent on loading conditions, was also obtained. The association between the presence of diastolic dysfunction and survival after lung transplantation in patients with pulmonary hypertension as the pathophysiological cause of left ventricular diastolic dysfunction was also shown [9,29]. Similarly, in this patient group, the use of ECMO was more frequent and the duration of ventilation was longer in the presence of diastolic dysfunction [9].

For the risk stratification of patients, not only the right ventricular but also the left ventricular function should be assessed, especially regarding diastolic dysfunction, already at the time of listing and preoperatively. The diastolic dysfunction can be excellently detected by echocardiography and also monitored intra- and post-operatively [30,31].

A specific evaluation of this pathology is not mentioned in the international recommendations. A cardiac assessment according to the recommendations of the American College of Cardiology (ACC) and the European Society of Cardiology (ESC) is recommended. This generally advises echocardiographic examinations including assessment of diastolic function in high-risk interventions and patients with evidence of clinically relevant cardiac disease, which includes the patient population in lung transplantation [14,16,17].

### 2.3. Reperfusion Injury

Post lung transplant oedema was described early as a complication after transplantation [32]. There is not always a correlation between radiological evidence of oedema and the severity of symptoms [33,34,35]. More recent views interpret reperfusion oedema with increased pulmonary vascular resistance alongside microvascular permeability disturbance, endothelial cell dysfunction, and sterile inflammation as RI [15]. Molecular and cellular mechanisms of this multi-layered complex syndrome are described in detail elsewhere [36]. Clinically, RI results in primary graft dysfunction (PGD) with fatal consequences for short- and long-term survival and morbidity after lung transplantation [37]. Pathophysiologically, it is now known that RI can be mitigated by the use of intraoperative circulatory support. As mentioned above, left ventricular deconditioning can cause diastolic dysfunction, which in turn exacerbates RI. The use of a VA ECMO intra- and post-operatively can provide the opportunity to unload the left ventricle and allow it to readapt stepwise to a normal CO [26]. Early analyses show an occurrence of oedema in more than half of the patients. Later data with all current therapeutic measures in both donors and recipients as well as in organ preservation suggest that an occurrence of RI in the context of PGD can be between 16% and 22%, also depending on the severity of the dysfunction [38,39].

Risk factors for the occurrence of RI with PGD and thus also reperfusion oedema can be divided into donor- and recipient-related factors. Nicotine abuse was found to be the most important donor risk factor. In addition, pre-mortem hypoxaemia, hypotension, aspiration, and prolonged mechanical ventilation are also known risk factors. Race, gender, and age also play a role. Recipient body mass index (BMI) and size mismatch are two of the most important receiver-related risk factors. In addition, high FiO_2_ during reperfusion, need for transfusion, and use of the heart-lung machine are noted to increase the risk for RI as intraoperative factors [40].

The consensus-based recommendations highlight the role of anaesthesiologists in the perioperative setting to avoid PGD, including reperfusion oedema. A general identification of modifiable risk factors that may influence the outcome after lung transplantation is recommended. Specific assessments for the identification of risk factors for PGD are not mentioned [14].

## 3. Strategy

In general, the strategy chosen for perioperative care in lung transplant patients varies greatly between the centres performing the operation. This is the result of an international survey conducted by Subramaniam et al. [41]. In our opinion, a comprehensive intraoperative strategy for circulatory support is based on adequate monitoring. Furthermore, the possibilities for non-mechanical and mechanical circulatory support are discussed in the following sections.

### 3.1. Monitoring

Intraoperative monitoring is the basis of every patient care strategy. New problems are detected and can be diagnosed. This section will focus on those aspects of monitoring that are relevant to circulatory support. Monitoring of anaesthesia depth, bronchoscopy, muscle relaxation, etc., are not included.

In principle, extended haemodynamic monitoring is recommended for the performance of lung transplantation. This includes an invasive blood pressure measurement in addition to the electrocardiogram (ECG), peripheral oxygen saturation (S_p_O_2_), and central venous pressure (CVP). Due to the frequent use of VA ECMO, cannulation of the right radial artery or the right brachial artery is recommended for this purpose [4,42]. In the case of poor oxygenation through the diseased lung or also the newly implanted lung and preserved pulsatility, there is the possibility that the endogenous blood flow with low oxygen content and the blood flow of VA ECMO with high oxygen content meet at the level of the aortic arch. Thus, it is possible, for example, that the oxygen content in the right carotid artery is too low, while there is no disturbance on the left side [43,44,45]. This phenomenon of the ECMO watershed and the possibly associated oxygen deficiency of the brain during the use of VA ECMO is attempted to be countered by monitoring the blood gases as close to the heart as possible. It also seems useful to monitor cerebral oxygenation and perfusion either by regular blood gas analyses or by using near-infrared spectroscopy (NIRS). Prospectively collected data suggest that acute brain damage can be detected in time. Pathophysiologically, the partial pressure of carbon dioxide (CO_2_) could play a role, as a reduction in cerebral blood flow can occur in the case of accidentally low CO_2_ levels under VA ECMO and mechanical ventilation. Especially in patients with permanently shifted CO_2_ partial pressures and chronic hypoxaemia, such as in advanced COPD, pathological changes in the autoregulation of cerebral blood flow are discussed [46]. In addition, monitoring of pulmonary arterial pressures and cardiac output will be added. PAP measurement can be used to derive indications for drug or mechanical afterload reduction for the right ventricle [42]. Pulmonary vascular resistance also plays a crucial role in the occurrence of RI and can contribute to early detection [15]. Intraoperative CO monitoring was introduced into anaesthesiologic management at the end of the 1990s and was initially associated with an improvement in mortality and morbidity [47]. Years later, this effect became somewhat less pronounced, which is probably due to the fact that some surgical methods are now much more minimally invasive [48]. Nevertheless, CO monitoring plays a decisive role in lung transplantation. It is particularly useful for assessing haemodynamic stability under single-lung ventilation and after pulmonary artery clamping, especially when no primary use of ECMO or CPB is planned. In some centres, it is a central component for establishing a secondary ECMO indication during transplantation [9].

Transoesophageal echocardiography (TOE) is another important component for assessing haemodynamics. It is one of the few types of monitoring that usually also allow a direct diagnosis of the underlying disease [49,50]. This plays a central role in the differential diagnosis between volume deficiency or pump failure or in identifying the cause of right heart failure. With regard to the assessment of right ventricular function, parameters in intraoperative use such as the assessment of RV size in relation to the LV, fractional area change (FAC), tricuspid annular systolic plane excursion (TAPSE), and septal motion have become established as valid procedures and are recommended accordingly [51]. The assessment of left ventricular function is also successful with TOE. Especially in view of the increasing focus on left ventricular diastolic dysfunction, repeated monitoring of the corresponding parameters is possible throughout the entire operation. The assessment is based on the E/e′ ratio [52]. It is worth noting that diastolic left ventricular dysfunction correlates with intrahospital mortality, but not systolic function [28]. In addition, TOE can be used to check the position of the guide wires and cannulae of extracorporeal support procedures, so that incorrect positions can be ruled out even before the onset of relevant complications. Cardiac pathologies such as a persistent foramen ovale can also be reliably detected by TOE. Thus, TOE has a decisive role in the context of lung transplantation. Even the examination of vascular anastomoses in the context of lung transplantation is possible and described in some case reports as relevant to therapy [12]. It is not surprising that TOE for lung transplantation is considered indispensable in the consensus-based recommendations for intraoperative management to assess haemodynamics [14].

### 3.2. Non-Mechanical Circulatory Support

Non-mechanical circulatory support can be divided into three main aspects: modulation of preload, myocardial contractility, and afterload.

In the perioperative care of patients undergoing high-risk interventions, there are various possibilities to enable targeted volume therapy. The assessment of the indication and the necessary amount for correct volume therapy can vary greatly between anaesthetists. Therefore, target-based treatment algorithms are generally recommended [53]. In principle, dynamic preload parameters best indicate whether volume administration is indicated or not [54]. This attempts to ensure optimal volume therapy, as both hypervolaemia and hypovolaemia are associated with negative effects for the patient [55]. In lung transplantation, however, the interpretation of these values is challenging, as the changing lung ventilation and the ongoing surgical manipulation in the thorax can distort the results of the measurement of dynamic preload parameters. Once again, TOE is an alternative to assess volume status. The correlation between high levels of CVP and poor outcome and the correlation between highly positive fluid balance and poor outcome are sufficiently proven [11,56]. Without restarting the long-lasting discussion about the choice of the most suitable volume replacement, it is recommended to use primarily crystalloid solutions and to use colloid solutions and blood products restrictively [57].

Various classes of drugs exist to increase the myocardial contractility. Among the typically used catecholamines, which exert their effect through the stimulation of ß-receptors, no drug could be shown to be clearly superior [58,59]. Even newer substance classes, so-called inodilators such as milrinone, showed no clear advantage over other drugs in prospective studies, despite the pulmonary vasodilatation by milrinone [60]. It should be mentioned that most of the studies were conducted in the context of cardiogenic shock and not in lung transplantation. The most common indication for myocardial contractility enhancement is RVF, for which there are also no robust and reliable data. In the experimental setting, epinephrine seems to be a good choice for the treatment of RVF, but clinically, no clear differences, e.g., to dobutamine, were demonstrated [61,62]. Individual case reports showed possible advantages for levosimendan. For example, the use of this drug led to better weaning of VA ECMO therapy in the context of RVF [63,64]. The administration of levosimendan may also lead to positive effects on left ventricular diastolic function. Recent studies showed that levosimendan acts by inhibiting phosphodiesterase and thus may contribute to pulmonary vasodilatation [65,66]. However, it should be kept in mind that positive inotropic substances often cause relevant side effects, which can be associated with a poorer clinical outcome [67,68]. Therefore, recommendations for the use of inotropics in lung transplantation are rather cautious [14].

To avoid systemic hypotension and the associated inadequate filling of the left ventricle in RVF, norepinephrine and vasopressin are the drugs of choice when used in the correct dose [14]. Vasopressin is even said to have a pulmonary vascular vasodilatation effect when used in low doses [69]. It has also been shown to reduce the need for other vasopressors during treatment of circulatory shock [70]. Finally, these two drugs play an important role in maintaining adequate perfusion pressure, especially when blood flow is adequately ensured by ECMO.

Inhaled nitric oxide (iNO) or prostacyclin are commonly used to reduce right ventricular afterload [12]. Intravenous sildenfil is also available as a therapeutic option [71]. The use of iNO in lung transplantation is very common internationally. Typical dosages are 10–20–40 ppm. Intraoperatively, iNO is used in 98% and postoperatively in 90%, of lung transplant cases [72]. The background is a dual effect of iNO. Inhaled NO can improve oxygenation by reducing the alveolar shunt volume and shows a pronounced reduction in right ventricular afterload without affecting systemic afterload [73]. Studies on the effects during transplantation demonstrated an improvement in oxygenation and a decrease in pulmonary arterial pressure [74]. However, randomised clinical data demonstrating an improvement in outcome are lacking. Effects on ICU length of stay, duration of ventilation, or time to extubation have not been proven beyond reasonable doubt [75]. A more recent study compared iNO with inhaled epoprostenol, which is significantly less expensive. There were no differences in outcome [74]. Side effects of iNO include renal failure, methaemoglobinaemia, and in the experimental setting, platelet dysfunction. However, the occurrence in adults is rare overall (<3%). Especially in lung transplantation, there was no higher incidence of renal failure observed [72,76]. Studies on the effect of iNO on RI showed contradictory results, so that overall, neither clinically nor currently a clear recommendation for or against iNO can be given on the basis of the available data [14,75]. This opinion is in line with current recommendations [14]. Here, iNO is favoured as a rescue therapy for RVF and prophylactic use is discouraged. In some centres, after the effect of iNO on right ventricular afterload has benefited from the reperfusion of the transplanted organ, an attempt is made to discontinue the application during the operation. iNO dependency appears to be a negative predictive factor for prognosis after lung transplantation [77]. Early data on the application of iloprost show beneficial effects for inhaled application [78]. This could also be confirmed in more recent clinical studies. Here, there was even a positive effect on the maintenance of a PGD, ICU LOS, and duration of ventilation [79]. The only disadvantage of iloprost as compared to iNO is the discontinuous application.

### 3.3. Mechanical Circulatory Support

Originally, CPB was used for mechanical circulatory support during lung transplantation [5]. In recent decades, ECMO has been increasingly used. This concept was developed from the first positive reports on the use of the procedure as bridging to transplant in the early 1990s [80]. With the improvement of technical conditions (polypentene fibre oxygenators, heparin-coated systems, etc.), the use of ECMO became more and more widespread. The existing prospective and observational data have so far been summarised in two meta-analyses [81,82]. Both articles conclude that the complication rate in terms of ventilation time, ICU LOS, and bleeding is better in the ECMO group as compared to the CPB group. Positive effects on short-term mortality were even demonstrated. Indications for CPB are the correction of a persisting foramen ovale (PFO) and an accidental massive blood loss during surgery [5].

In view of the positive effects of ECMO as compared to CPB and the pathophysiological consideration of gradually adapting the deconditioned left ventricle to normal CO values, strategies have also been described for using VA ECMO in 100% of cases of lung transplantation. Hoetzenecker et al. were able to achieve the lowest rates of PGD after lung transplantation to date with this regimen [10]. Other high-volume centres report rates of 15–28% for the use of intraoperative ECMO [5,8]. Here, a risk-adjusted approach is favoured. Patients with idiopathic pulmonary fibrosis, marked PAH, and left ventricular diastolic dysfunction are candidates for ECMO. Other indications result from the intraoperative course. Especially after clamping of the pulmonary arteries, the indication for VA ECMO is made liberally in the case of haemodynamic relevance (see Table 2). Additionally, there is some evidence that utilization of ECMO relates to the type of donor. Sef et al. reported a more common intraoperative mechanical circulatory support, when organs were donated after circulatory death as compared to donation after brain death [83]. Overall, vascular complication rates of 2–25% have been described for intraoperative use of ECMO. Increased occurrence of PGD, renal replacement therapy, longer ICU LOS, higher fluid balance, and even a higher short-term mortality rate have also been described with the use of ECMO as compared to conventional surgery [9]. However, as these are retrospectively evaluated data, a selection bias cannot be ruled out.

The question of the ideal cannulation strategy cannot be answered so simply. Of course, this has to be based on the patient’s individual requirements. Classically, an attempt is made to establish peripheral cannulation via the femoral vessels. The advantages here are the low invasiveness and easy removal. However, there are also disadvantages of this access. Perfusion of the leg is limited or must be ensured by an additional cannula in the femoral artery. If the oxygenation capacity of the perfused lung is limited, there is a risk of cerebral oxygen deficiency in the context of the occurrence of an ECMO watershed [43]. This can be circumvented by central cannulation into the ascending aorta. However, this also requires a full sternotomy [84]. In addition, a central ECMO must be surgically removed and complicates mobilisation in the ICU. These drawbacks can be avoided by inserting a VVA ECMO or connecting the arterial cannula to the subclavian artery using a prothesis [3,5,7].

Overall, the use of VA ECMO is superior to CPB in most cases. Especially in severely pre-diseased patients, the use of VA ECMO is more advantageous as compared to a CPB approach or even non-mechanical circulatory support. However, this also requires a consistent ECMO weaning programme and concept to prevent long ventilation times and longer ICU stays.

## 4. Discussion

This article summarises potential options for intraoperative circulatory support in lung transplantation. We have reviewed risk factors for three exemplary but frequent haemodynamic complications as well as possibilities for all types of circulatory support.

Pre-existing intermediate and severe PAH and idiopathic pulmonary fibrosis are particular risk factors for the occurrence of RVF preoperatively [5,12,85]. Intraoperatively, echocardiographic signs of acute (on chronic) right ventricular strain and suprasystemic pulmonary arterial pressures are indicators that make right ventricular decompensation likely. Left ventricular diastolic dysfunction is becoming increasingly important in terms of poor outcome after lung transplantation according to recent pathophysiological findings [26]. Echocardiographic assessment prior to surgery has a high predictive value to identify patients at risk for LVF due to diastolic dysfunction [86]. However, the administration of colloid fluids was one of the few independent variables in the perioperative management besides the use of CPB and a high inspiratory oxygen fraction, which led to an increased occurrence of PGD [87]. Risk factors for PGD that are already identifiable in advance are donor-related hypoxaemia, hypotension, aspiration, and prolonged mechanical ventilation. Recipient-related risk factors are a transplant size mismatch and recipient BMI [40] (see Table 1).

After recognising risk factors (depending on the individual patient), it is crucial to develop an appropriate therapeutic strategy for the best possible circulatory support with the least complications. The consensus-based recommendations for anaesthesiologic and intensive care management in lung transplantation emphasise the role of the anaesthesiologist as an integral member of the multidisciplinary team caring for those patients. After appropriate screening for risk factors related to very different complications, jointly developed management is also recommended [14].

Assessment of sufficiency of non-mechanical circulatory support is crucial in the perioperative phase. Using various monitoring methods, it is important to avoid both hyper- and hypo-volaemia [55]. The measurement of pulmonary arterial pressures and CO as well as TOE continues to be of particular importance [4,44]. There is a lack of robust data to definitively recommend specific catecholamine therapies. However, levosimendan seems to be a useful and promising option in selected cases [88,89]. To reduce RV afterload, the inhalation of NO or iloprost has equal effects [75]. If possible, intraoperative weaning should be attempted. If mechanical circulatory support is indicated, VA ECMO is preferable to CPB [81]. High survival rates are also described for postoperative use of VA ECMO [90].

Ultimately, the question must be asked whether the implementation of an early risk stratification and strategy (ERSAS, see Figure 1) algorithm, i.e., haemodynamic intraoperative management in general, is relevant to the outcome. Results from a study investigating the impact of the anaesthesiologic management on PGD showed that the administration of colloid fluids was an independent risk factor for the occurrence of PGD [57]. Furthermore, the choice of the right haemodynamic management may be crucial [10,26]. Hoetzenecker et al. reported the lowest rate of PGD using the haemodynamic approach with VA ECMO according to their departmental standard operating procedure. Tudorache et al. showed that the need for secondary ECMO as a rescue therapy was one of the few factors that influenced intrahospital mortality in a multivariate analysis. In this setting, the use of secondary ECMO potentially suggests a haemodynamic situation that had not been adequately controlled [9].

In this context, the data from an international survey on lung transplant management are interesting [41]. Here, large differences between the hospitals performing the transplantation could be shown. Almost 50% of included centres had no protocol for catecholamine therapy, 87% used TOE intraoperatively, but only 63% monitored pulmonary arterial pressure. The authors interpreted the large variance as possible gaps in care and knowledge and identified opportunities for improvement in clinical care.

Finally, only selected literature can be reproduced in such an article. Thus, selection bias and centre-based views are notable limitations. The quantity and quality of data on the management of lung transplantation are also limited by the small number of performing centres and the rarity of the procedure.

## Figures and Tables

**Table 1:** Summary of selected risk factors for haemodynamic complications.

Haemodynamic Complications	Risk Factors	Tool for Determination
RVF	PAH	PAC, TOE
	tolerance of PA clamping	haemodynamic monitoring
LVF	physical resilience	general preoperative assessments
	E/e′	TTE, TOE
RI	donor: hypoxia, hypotension, aspiration, ischemic time	BGA, monitoring, CT scan
	recipient: size mismatch, BMI	general assessments
	amount colloidal volume replacement	protocol based intraoperative care

RVF: right ventricular failure; PAH: pulmonal arterial hypertension; PAC: pulmonal artery catheter; TOE: transoesophageal echocardiography; LVF: left ventricular failure; E/e′: ratio of early transmitral flow (E) and mitral annular velocity (e′); TTE: transthoracic echocardiography; RI: reperfusion injury; BGA: blood gas analysis; BMI: body mass index.

**Table 2:** Indications for mechanical circulatory support.

Indication	Parameter	Time of Onset
idiopathic pulmonary fibrosis	not applicable	preoperative
intermediate to severe PAH	systemic and suprasystemic PAP	pre- and intra-operative
acute on chronic RVF	increased RVEDD poor RV contractility	after PA clamping
acute LVF	CI < 2 L/min/m^2^	after reperfusion
impaired gas exchange	hypercapnia/hypoxia	intra- and post-operative
insufficient non-mechanical circulatory support	increasing need for vasopressors and/or inotorpes decreased CI and/or S_cv_O_2_	any time during treatement

PAP: pulmonal arterial pressure; PA: pulmonal artery; RVEDD: right ventricular end diastolic diameter; RV: right ventricular; CI: cardiac index; S_cv_O_2_: central venous oxygen saturation.
